# The oncogenic role of SNRPB in human tumors: A pan-cancer analysis

**DOI:** 10.3389/fmolb.2022.994440

**Published:** 2022-10-06

**Authors:** Juan Wu, Feng Lu, Bin Yu, Wenjun Wang, Xiaoqun Ye

**Affiliations:** ^1^ Department of Respiratory Diseases, The Second Affiliated Hospital of Nanchang University, Nanchang, China; ^2^ Department of Cardiothoracic Surgery, The Second Affiliated Hospital of Nanchang University, Nanchang, China

**Keywords:** SNRPB, small nuclear ribonucleoprotein polypeptides B and B1, pan-cancer, human tumors, oncogenic role, oncogene

## Abstract

**Purpose:** The purpose of this study was to explore the oncogenic role of small nuclear ribonucleoprotein polypeptides B and B1 (SNRPB) in human tumors.

**Materials and methods:** Study cases were acquired from The Cancer Genome Atlas database, the Gene Expression Omnibus database, The Human Protein Atlas, and the Clinical Proteomic Tumor Analysis Consortium. We then used the R package and several online tools to analyze and visualize the role of SNRPB across tumors.

**Results:** We found that the expression of SNRPB was significantly increased in 28 of 33 tumors, and higher expression was observed in late pathological and TNM stages. Significantly decreased levels of SNRPB promoter methylation were observed in 12 tumors. SNRPB was found to be a risk factor for decreased overall survival in 10 tumors (*p* < 0.05), a risk factor for decreased disease-specific survival in 8 tumors (*p* < 0.05), and a risk factor for decreased progression-free interval in 7 tumors (*p* < 0.05). The PPI network of SNRPB and the top 100 coexpressed genes revealed that CDK1, CDC6, AURKB, CCNB1, CCNA2, and CDC45 were the most closely interacting genes across tumors. The GO and KEGG enrichment analyses revealed that SNRPB and the above genes were mainly enriched with respect to functions in cell cycle-related genetic material replication, assembly, and distribution. SNRPB was significantly associated with immune cell infiltration and the expression of immunomodulation-related genes in several but not all tumors.

**Conclusion and limitations:** The expression of SNRPB was significantly elevated in almost all tumors, and the decreased promoter methylation level may contribute to the elevated expression of SNRPB. SNRPB may facilitate the progression of pathological and TNM stages and is a risk factor for unfavorable prognosis across tumors. However, our research was based on data obtained from public databases, without further validation of our findings at the cellular and animal levels. Therefore, further studies are needed to clarify the oncogenic mechanism of SNRPB and its potential as a therapeutic target.

## Introduction

As one of the deadliest diseases in the world, cancer is the leading cause of premature death (Bray et al., 2021). Along with the transition of cells from normal to neoplastic states, they acquire various functions necessary for their malignancy, including persistent proliferation signals, genome instability and mutation, immune escape, invasion and metastasis, unlocked phenotypic plasticity, nonmutational epigenetic reprogramming, and the activation of oncogenes (Hanahan and Weinberg, 2011; Hanahan, 2022). The identification of oncogenes and their roles across the spectrum of human cancers is of great importance to better understand the complex pathological mechanism of cancers.

The alternative splicing (AS) of most human genes gives rise to transcript “isoforms” (Pan et al., 2008; Wang et al., 2008), increases the diversity of mRNA expression, and results in functional diversity of the encoded proteins based on enzymatic activity, subcellular localization, and protein‒ligand, protein‒protein, and protein‒DNA physical interactions (Kelemen et al., 2013), which have profound effects on the proliferation and survival of cells (Kelemen et al., 2013). Spliceosomes are required for AS (Scofield and Lynch, 2008; Liu et al., 2019).

Small nuclear ribonucleoprotein polypeptide B and B1 (SNRPB) is a core component of the spliceosome and thus plays a critical role in pre-mRNA splicing (Gray et al., 1999). Dysregulation of SNRPB influences the splicing of pre-mRNA and generates unexpected mRNA variants. The protein translated by these new mRNA variants may play an important role in tumorigenesis (Correa et al., 2016; Liu et al., 2019; Peng et al., 2020; Zhan et al., 2020). The role of SNRPB in promoting tumorigenesis and progression has been observed in tumors such as non-small cell lung cancer (NSCLC) (Liu et al., 2019; Liu et al., 2021), hepatocellular carcinoma (LIHC) (Peng et al., 2020; Zhan et al., 2020; Li et al., 2022), glioblastoma (GBM) (Correa et al., 2016), cervical cancer (CESC) (Zhu et al., 2020), and thyroid carcinoma (THCA) (Deng et al., 2022). However, the role of SNRPB in other tumors remains unclear.

To comprehensively explore the oncogenic role of SNRPB in human tumors, we acquired study cases from The Cancer Genome Atlas database (TCGA), the Gene Expression Omnibus database (GEO) (Barrett et al., 2013), The Human Protein Atlas (HPA) (Uhlen et al., 2017), and the Clinical Proteomic Tumor Analysis Consortium (CPTAC) dataset from the University of ALabama at Birmingham CANcer data analysis portal (Chandrashekar et al., 2017) (UALCAN). We then performed an in-depth pan-cancer analysis of SNRPB on the mRNA and protein expression, prognostic value, genetic variation, promoter methylation, the possible oncogenic mechanism, and the immunological role, which would provide us with new ideas and a theoretical basis for cancer diagnosis and treatment.

## Materials and methods

### Data source and preparation

Case information about mRNA expression (normalized as transcripts per million reads, TPM) and clinical features was obtained from TCGA and Genotype-Tissue Expression project (Consortium, 2013) (GTEx) and downloaded from the University of California Santa Cruz Xena (Goldman et al., 2020) (UCSC Xena, https://xena.ucsc.edu/) platform. Microarray data were downloaded from GEO database (Barrett et al., 2013) (http://www.ncbi.nlm.nih.gov/geo/). The raw data were downloaded as MINiML files and were normalized by log2 transformation. We used the normalize quantiles function of the preprocessCore package in R to normalize the microarray data. Probes were converted to gene symbols according to the annotation of the normalized data in the platform. RemoveBatchEffect function of the limma package in R was used to remove batch effect of samples in different batches. Boxplot was used to assess the result of the data preprocessing. All data in Supplementary Figure S1 were comparable after normalization (the boxplots of data preprocessing results were not shown). Protein expression data were obtained from UALCAN data analysis portal (Chandrashekar et al., 2017) (http://ualcan.path.uab.edu/analysis-prot.html, CPTAC dataset). Immunohistochemistry (IHC)-based protein expression patterns were acquired from HPA (Uhlen et al., 2017) (Human Protein Atlas proteinatlas.org). Genetic alteration data were obtained from cBioPortal (Cerami et al., 2012) (http://www.cbioportal.org). Promoter methylation data were obtained from UALCAN data analysis portal (TCGA dataset). The top 100 coexpressed genes were obtained from Gene Expression Profiling Interactive Analysis (Tang et al., 2019) (GEPIA, version 2, http://gepia2. cancer-pku.cn/#index). Protein‒protein interaction (PPI) network were obtained from STRING (Szklarczyk et al., 2021) (https://string-db.org/). Data regarding the relationship between SNRPB and immune cell infiltration as well as immunomodulation-related gene expression were obtained from the Tumor Immune Estimation Resource (Li et al., 2020) (TIMER, version 2, timer.cistrome.org).

### mRNA and protein expression of SNRPB across tumors

We first used the Wilcoxon test to compare the expression level of SNRPB in tumors and the corresponding paracancerous tissues in TIMER2. Since there were no corresponding paracancerous tissue data for some tumors in TCGA, we used data obtained from TCGA and GTEx to compare the expression level of SNRPB in tumors and corresponding normal tissues. The “ggplot2” R package (version 3.3.3) was used to analyze and visualize the results, and an unpaired samples *t* test was used to compare the expression level of SNRPB between the normal and tumor groups. Normalized SNRPB expression data from GEO were compared using the Wilcoxon test.

Protein expression data of primary tumors and the corresponding normal tissues in the CPTAC dataset from UALCAN data analysis portal were compared.

### IHC-based protein expression of SNRPB across tumors

To verify the protein expression of SNRPB at the histological level, IHC-based protein expression patterns in bladder urothelial carcinoma (BLCA), breast invasive carcinoma (BRCA), CESC, colon adenocarcinoma (COAD), LIHC, lung adenocarcinoma (LUAD), lung squamous cell carcinoma (LUSC), ovarian serous cystadenocarcinoma (OV), pancreatic adenocarcinoma (PAAD), prostate adenocarcinoma (PRAD), skin cutaneous melanoma (SKCM), stomach adenocarcinoma (STAD), testicular germ cell tumors (TGCT), uterine corpus endometrial carcinoma (UCEC), and the corresponding normal tissues were obtained from HPA.

### mRNA and protein expression of SNRPB in different pathological stages across tumors

GEPIA2 was used to assess the correlation between SNRPB mRNA expression and pathological stages; CPTAC samples from UALCAN data analysis portal were used to assess the correlation between SNRPB protein expression and pathological stages.

### SNRPB expression and TNM stages

The clinical datasets obtained from TCGA were used to explore the effect of SNRPB expression on TNM stages. With 50% as the cutoff value, samples were divided into low and high groups.

### Survival and prognostic analysis

The clinical datasets obtained from TCGA were used to perform the survival and prognostic analysis. With 50% as the cutoff value, samples were divided into low and high groups. Overall survival (OS), disease-specific survival (DSS), and progression-free interval (PFI) were used for the evaluation of survival and prognostic outcomes. We performed Kaplan‒Meier (KM) analysis with Cox regression using the “survminer” and “survival” packages in R.

### Genetic alteration

The “TCGA PanCancer Atlas Studies” in cBioPortal were used to analyze and visualize the genetic alteration of SNRPB and the impact of genetic alteration on OS, disease-free survival (DFS), DSS, and progression-free survival (PFS) across tumors.

### Promoter methylation level of SNRPB across tumors

TCGA samples from UALCAN data analysis portal were used to compare the promoter methylation level of SNRPB between primary tumors and the corresponding normal tissues.

### Coexpressed genes and PPI network

The top 100 coexpressed genes of SNRPB were obtained from GEPIA2 based on the datasets of all TCGA tumors. The “Correlation Analysis” module in GEPIA2 was used to analyze and visualize the correlation between SNRPB and the top 6 coexpressed genes, and Pearson correlation was used in the above analysis. The correlation heatmap of SNRPB and the top 10 coexpressed genes was analyzed and plotted using TIMER2. To explore the proteins closely interacting with SNRPB across tumors, we used STRING (https://cn.string-db.org; main parameters: network type: full STRING network, meaning of network edges: evidence, active interaction source: Textmining, Experiments, Databases, Co-expression, Neighborhood, Gene Fusion, and Co-occurrence, minimum required interaction score: Medium confidence [0.400], max number of interactors to show: 1st shell [no more than 50 interactors], 2nd shell [none/query proteins only]) to analyze the relationship between the proteins expressed by SNRPB and the top 100 coexpressed genes and visualized them using Cytoscape (Shannon et al., 2003).

### Functional annotation

SNRPB and the top 100 coexpressed genes obtained from GEPIA2 (101 genes in total) were used to perform Gene Ontology (GO) and Kyoto Encyclopedia of Genes and Genomes (KEGG) enrichment analyses in R using the “ggplot2”, “clusterProfiler”, and “GOplot” packages.

### Immune infiltration analysis

We first used the TIMER algorithm to assess the relationship between SNRPB and the infiltration of CD8^+^ T cells, CD4^+^ T cells, B cells, macrophages, neutrophils, and dendritic cells. Then, the xCell algorithm was used to evaluate the relationship between SNRPB and the infiltration of immune cell subtypes in TIMER2. The correlation between SNRPB and the expression of immunomodulation-related genes across tumors was also analyzed in TIMER2. Finally, we visualized them in R using the “ggplot2” package.

## Results

### SNRPB expression was significantly elevated in almost all tumors

**FIGURE 1:**
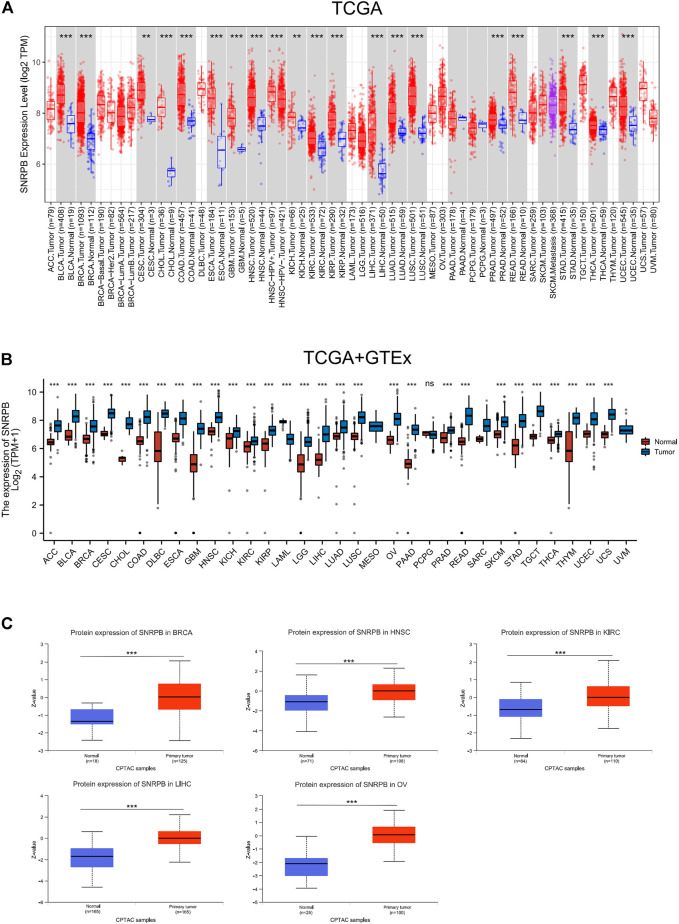
mRNA and protein expression levels of SNRPB across tumors. **(A)** mRNA expression level of SNRPB in tumors and adjacent paracancerous tissues in TCGA from TIMER2. **(B)** mRNA expression level of SNRPB in tumors and the corresponding normal tissues in TCGA and GTEx. **(C)**. Protein expression level of SNRPB in primary tumors and the corresponding normal tissues in the CTPAC dataset from UALCAN. ns, no significant difference; *, *p* < 0.05; **, *p* < 0.01; ***, *p* < 0.001.

A total of 21 tumors in the TCGA database had data on corresponding paracancerous tissues, and the expression of SNRPB was significantly elevated in 19 of them (*p* < 0.05, Figure 1A). Since there were no corresponding paracancerous tissue data for some tumors in TCGA, we used data obtained from TCGA and GTEx to compare the expression level of SNRPB in cancer and corresponding normal tissues, and the expression of SNRPB was found to be significantly increased in 28 of 33 tumors (*p* < 0.05, Figure 1B). To further clarify the expression of SNRPB across tumors, we obtained the expression levels of SNRPB in 21 tumors from the GEO database, and the expression of SNRPB was found to be significantly increased in 17 of 21 tumors (*p* < 0.05, Supplementary Figure S1).

**FIGURE 2:**
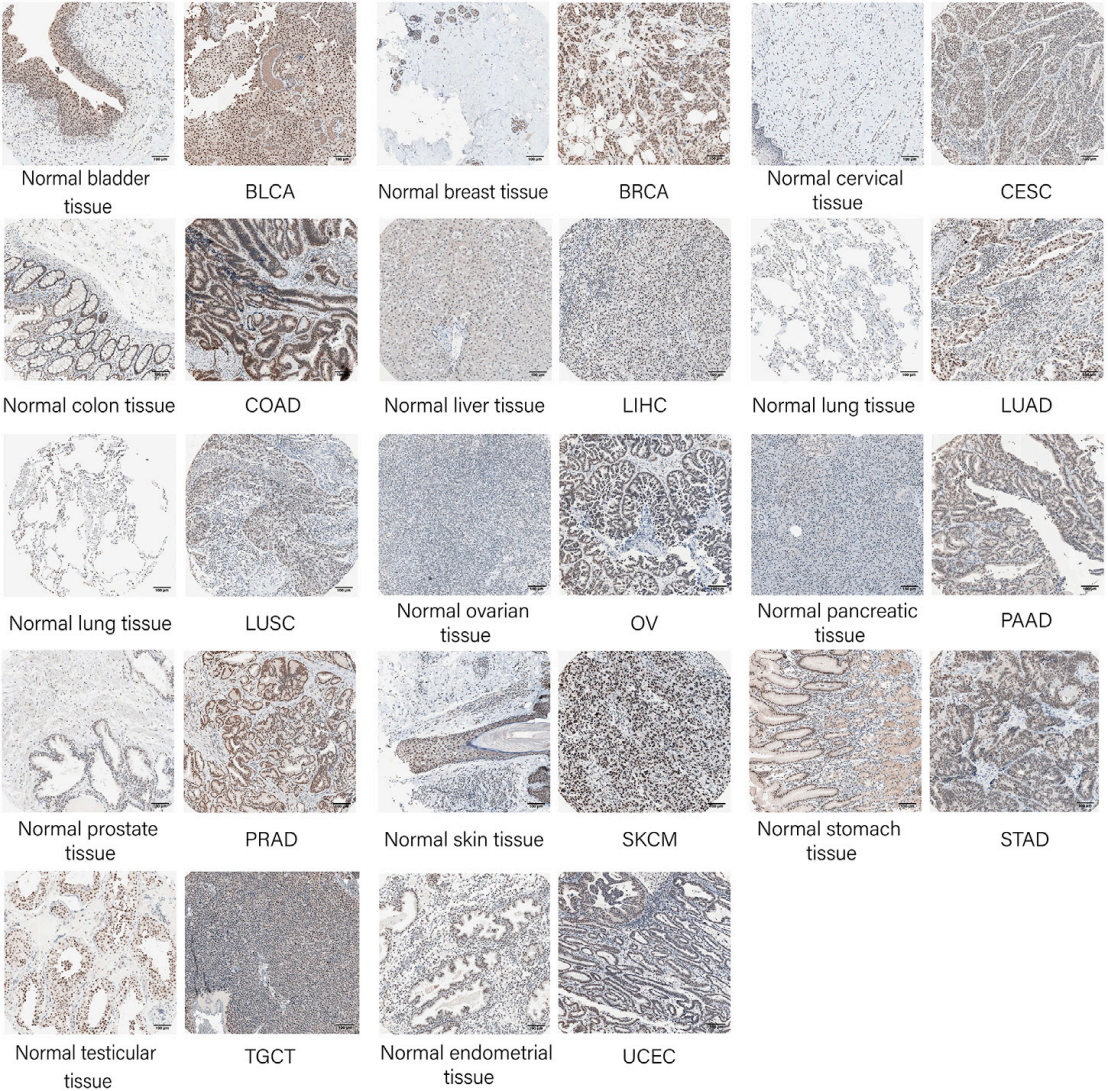
Validation of SNRPB protein expression in tumors and the corresponding normal tissues in HPA.

To verify the protein expression level of SNRPB across tumors, we first acquired protein expression data of primary tumors and the corresponding normal tissues in the CPTAC dataset from UALCAN data analysis portal. Only limited protein expression data of SNRPB were available, including BRCA, head and neck squamous cell carcinoma (HNSC), kidney renal clear cell carcinoma (KIRC), LIHC, and OV, and the protein expression level of SNRPB was significantly increased in all five tumors (Figure 1C, *p* < 0.05). Then, IHC-based protein expression patterns in BLCA, BRCA, CESC, COAD, LIHC, LUAD, LUSC, OV, PAAD, PRAD, SKCM, STAD, TGCT, UCEC, and the corresponding normal tissues were obtained from HPA. Compared with normal tissues, the IHC-based SNRPB expression level was significantly increased in the above 14 tumors (Figure 2).

### The expression of SNRPB was higher in late pathological stages

**FIGURE 3:**
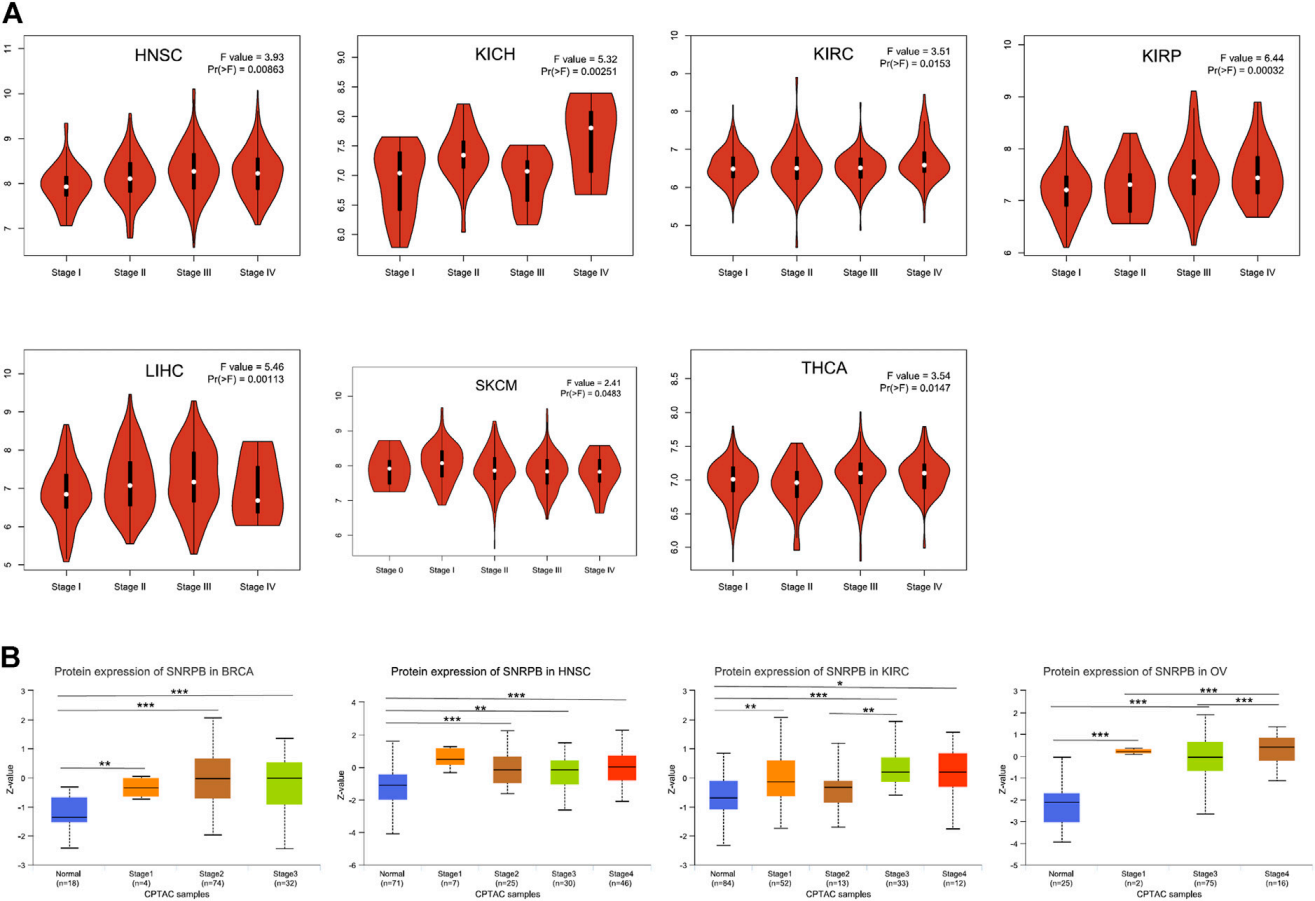
mRNA and protein expression levels of SNRPB in different pathological stages across tumors. **(A)** mRNA expression level of SNRPB in different pathological stages. **(B)**. Protein expression level of SNRPB in different pathological stages.*, *p* < 0.05; **, *p* < 0.01; ***, *p* < 0.001.

To identify whether SNRPB is differentially expressed among pathological stages, we first analyzed the correlations between the mRNA expression of SNRPB and the pathological stages across tumors using GEPIA2. The results showed that SNRPB was differentially expressed among pathological stages of HNSC, kidney chromophobe (KICH), KIRC, kidney renal papillary cell carcinoma (KIRP), LIHC, SKCM, and THCA, and the expression levels were generally higher in late pathological stages (*p* < 0.05, Figure 3A). Then, SNRPB protein expression data of the CPTAC dataset from UALCAN data analysis portal were obtained, and significantly differential expression of SNRPB was observed in BRCA, HNSC, KIRC, and OV. Additionally, the protein expression levels were generally higher in late pathological stages (*p* < 0.05, Figure 3B). Thus, SNRPB may promote the progression of pathological stages across tumors.

### SNRPB may facilitate the progression of TNM stages

The differential expression pattern of SNRPB in pathological stages suggests that SNRPB may also promote the progression of TNM stages. To explore the relationship between SNRPB and TNM stages, we compared the differences in T (T1, T2, T3, T4), N (N0, N1, N2, N3), and M (M0, M1) stages between the low and high groups. As shown in Table 1, compared with the low group, the proportion of T1 (T2 in PRAD) stage was lower, whereas those of T2 (in BRCA, KIRP, LIHC), T3 (in BRCA, KIRP, LIHC, and PRAD) and T4 (in LIHC and PRAD) stages were higher in the high group. The same phenomenon was also observed for the N and M stages. As shown in Table 2, compared with the low group, the proportion of the N0 stage was lower, whereas N1, N2, and N3 stages were higher in the high group in HNSC, KIRP (only N1 and N2 stages), and LUAD. As shown in Table 3, compared with the low group, the proportion of M0 (except for LUAD) stage was lower, whereas the M1 stage was higher in the high group in BLCA, KIRP, LUAD, and rectum adenocarcinoma (READ). The above results suggest that the expression of SNRPB may promote local tumor progression, lymph node metastasis, and distant metastasis, thus facilitating the progression of TNM stages.

**TABLE 1:** The relationship between SNRPB expression and tumor T stages.

Tumor	SNRPB expression level	T1 (n [%])	T2 (n [%])	T3 (n [%])	T4 (n [%])	*p* Value	Method
BRCA						0.005	Chi-square test
	Low (*n* = 541)	160 (14.8%)	296 (27.4%)	62 (5.7%)	22 (2%)		
	High (*n* = 542)	117 (10.8%)	333 (30.8%)	77 (7.1%)	13 (1.2%)		
KIRP						0.020	Fisher’s exact test
	Low (*n* = 144)	107 (37.3%)	13 (4.5%)	21 (7.3%)	1 (0.3%)		
	High (*n* = 145)	86 (30%)	20 (7%)	38 (13.2%)	1 (0.3%)		
LIHC						0.010	Chi-square test
	Low (*n* = 187)	107 (28.8%)	40 (10.8%)	34 (9.2%)	4 (1.1%)		
	High (*n* = 187)	76 (20.5%)	55 (14.8%)	46 (12.4%)	9 (2.4%)		
PRAD						0.004	Chi-square test
	Low (*n* = 249)	-	111 (22.6%)	131 (26.6%)	3 (0.6%)		
	High (*n* = 250)	-	78 (15.9%)	161 (32.7%)	8 (1.6%)		

**TABLE 2:** The relationship between SNRPB expression and tumor N stages.

Tumor	SNRPB expression level	N0 (n [%])	N1 (n [%])	N2 (n [%])	N3 (n [%])	*p* Value	Method
HNSC						0.002	Fisher’s exact test
	Low (*n* = 251)	138 (28.7%)	37 (7.7%)	63 (13.1%)	1 (0.2%)		
	High (*n* = 251)	101 (21%)	43 (9%)	91 (19%)	6 (1.2%)		
KIRP						0.017	Fisher’s exact test
	Low (*n* = 144)	26 (33.8%)	5 (6.5%)	1 (1.3%)	-		
	High (*n* = 145)	23 (29.9%)	19 (24.7%)	3 (3.9%)	-		
LUAD						0.017	Fisher’s exact test
	Low (*n* = 267)	185 (35.6%)	36 (6.9%)	33 (6.4%)	0 (0%)		
	High (*n* = 268)	163 (31.4%)	59 (11.4%)	41 (7.9%)	2 (0.4%)		

**TABLE 3:** The relationship between SNRPB expression and tumor M stages.

Tumor	SNRPB expression level	M0 (n [%])	M1 (n [%])	*p* Value	Method
BLCA				0.011	Fisher’s exact test
	Low (*n* = 207)	118 (55.4%)	2 (0.9%)		
	High (*n* = 207)	84 (39.4%)	9 (4.2%)		
KIRP				0.012	Fisher’s exact test
	Low (*n* = 144)	54 (51.9%)	1 (1%)		
	High (*n* = 145)	41 (39.4%)	8 (7.7%)		
LUAD				0.013	Chi-square test
	Low (*n* = 267)	172 (44.6%)	5 (1.3%)		
	High (*n* = 268)	189 (49%)	20 (5.2%)		
READ				0.013	Chi-square test
	Low (*n* = 83)	66 (44.3%)	5 (3.4%)		
	High (*n* = 83)	60 (40.3%)	18 (12.1%)		

### SNRPB was a risk factor for unfavorable prognosis across tumors

We used OS, DSS and PFI as outcomes to assess the survival and prognostic value of SNRPB across tumors. To show the results more clearly and concisely, we first generated a forest plot of the prognostic value of SNRPB across tumors and then displayed the KM curve plot of tumors in which SNRPB had a significant impact on prognosis on the right side of the forest plot.

**FIGURE 4:**
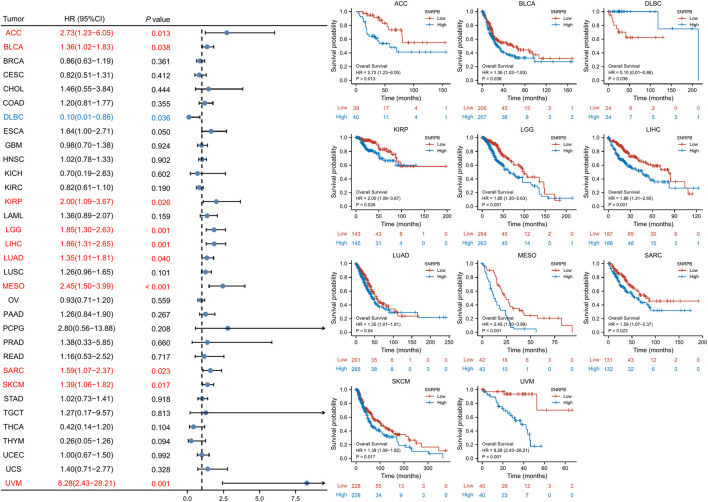
The relationship between SNRPB expression and OS across tumors.

**FIGURE 5:**
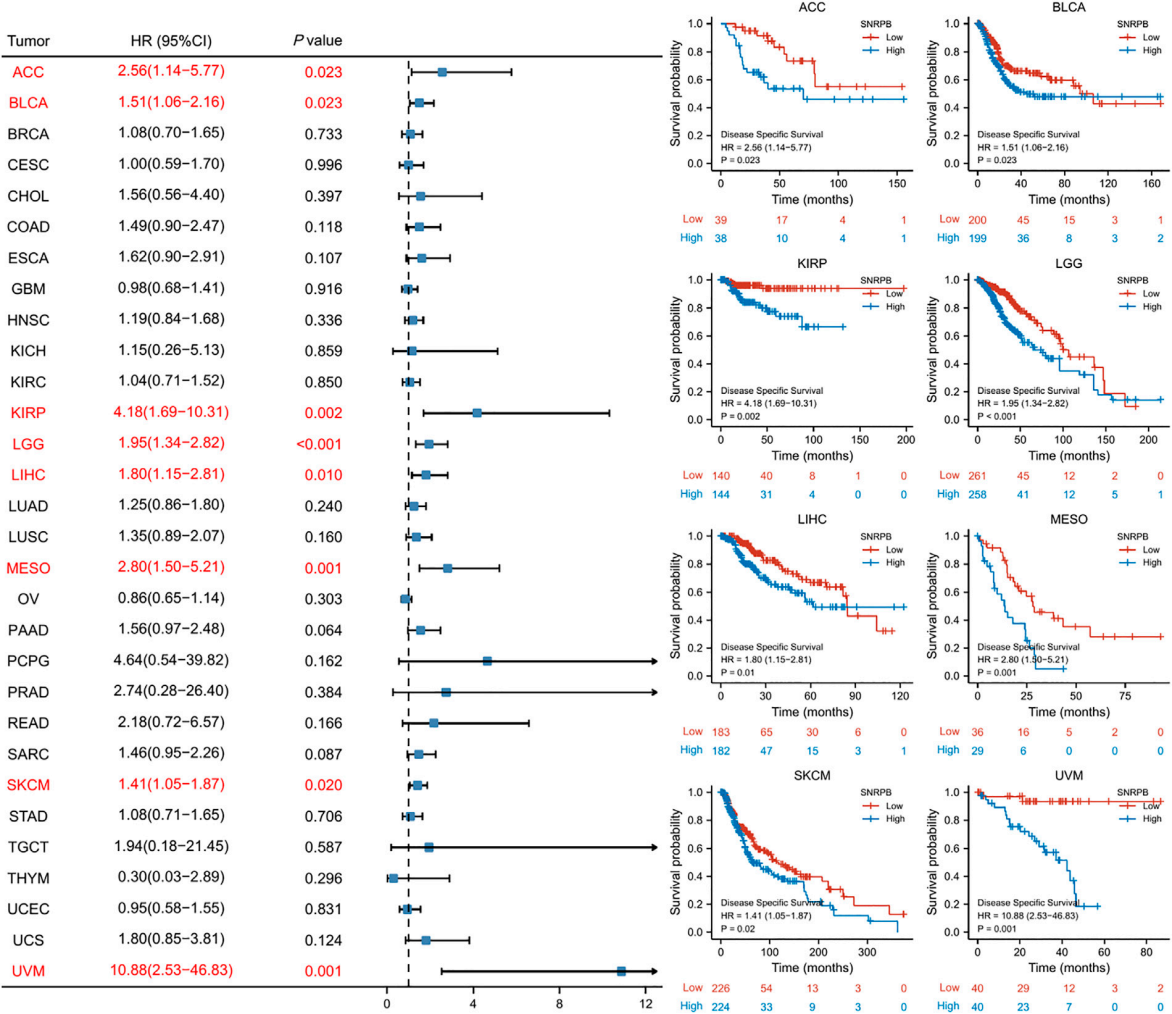
The relationship between SNRPB expression and DSS across tumors.

**FIGURE 6:**
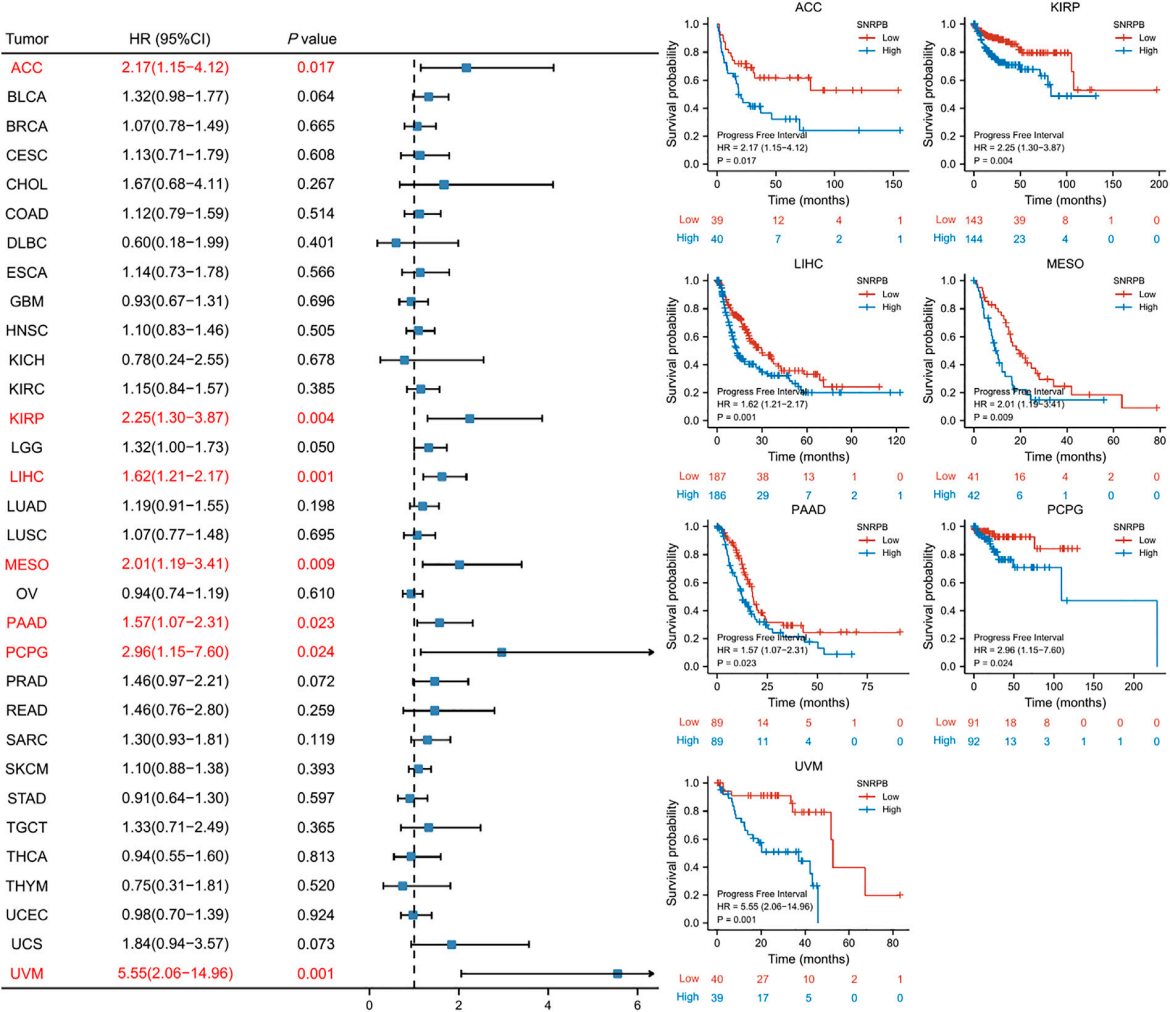
The relationship between SNRPB expression and PFI across tumors.

The results showed that high expression of SNRPB was a risk factor for unfavorable prognosis in several tumors. As shown in Figure 4, SNRPB was found to be a risk factor for decreased OS in 10 tumors, including adrenocortical carcinoma (ACC), BLCA, KIRP, brain lower grade glioma (LGG), LIHC, LUAD, mesothelioma (MESO), sarcoma (SARC), SKCM, and uveal melanoma (UVM) (*p* < 0.05). As shown in Figure 5, SNRPB was found to be a risk factor for decreased DSS in 8 tumors, including ACC, BLCA, KIRP, LGG, LIHC, MESO, SKCM, and UVM (*p* < 0.05). As shown in Figure 6, SNRPB was found to be a risk factor for decreased PFI in 7 tumors, including ACC, KIRP, LIHC, MESO, PAAD, pheochromocytoma and paraganglioma (PCPG), and UVM (*p* < 0.05). Therefore, high SNRPB expression could be a risk factor for unfavorable prognosis across tumors.

### Genetic alteration of SNRPB across tumors

**FIGURE 7:**
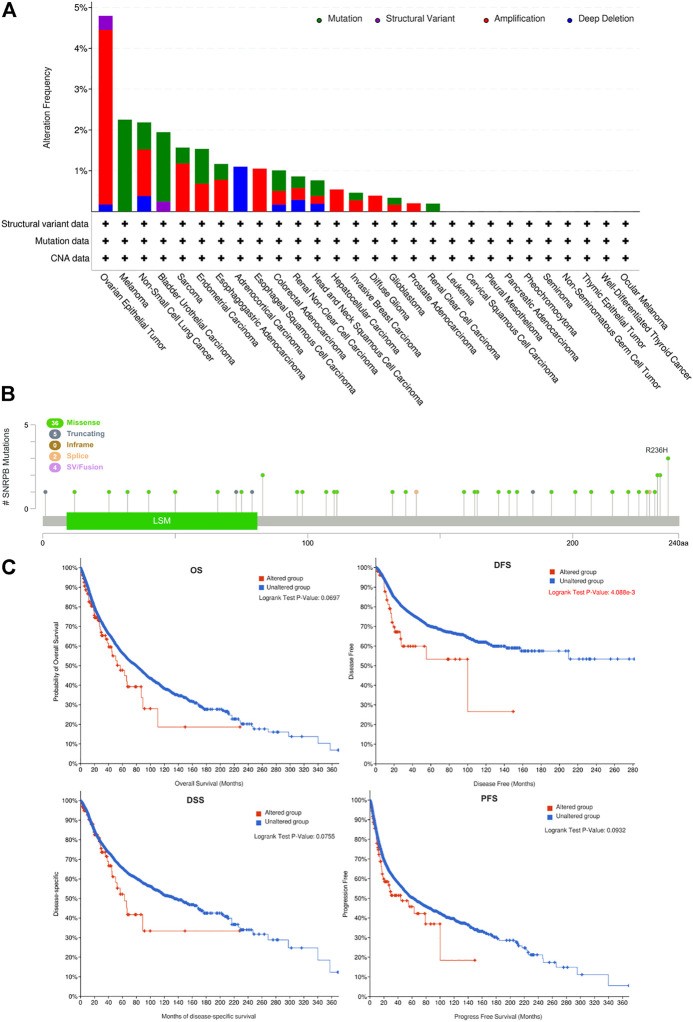
Genetic alteration of SNRPB across tumors. **(A)**. Summary of genetic alterations of SNRPB across tumors. **(B)**. Mutated SNRPB sites across tumors. **(C)**. The effect of genetic alteration on OS, DFS, DSS, and PFS across tumors.

Genetic alterations may affect gene expression levels and their prognostic role in tumors (Yang et al., 2019; Zhang et al., 2020). To explore the genetic alteration and its prognostic role across tumors, we obtained SNRPB gene variation data from cBioPortal. The results showed that amplification and mutation are the most frequent alterations across tumors. OV was the tumor with the highest mutation rate, and 4.79% (28/584) of the OV cases harbored genetic variations (Figure 7A). We then investigated the main mutation types and their locations within SNRPB. Missense mutations were the most common mutation type and were randomly distributed within SNRPB, and R236H was the most mutated site, where 3 missense mutations occurred (Figure 7B). Finally, we evaluated the effect of SNRPB genetic alterations on prognosis across tumors (Figure 7C) and found that DFS in the altered group was significantly lower than that in the unaltered group (*p* < 0.05). However, no significant effect of genetic alteration was observed on OS, DSS, or PFS across tumors (*p* > 0.05).

### Decreased promoter methylation levels may contribute to the elevated expression of SNRPB across tumors

**FIGURE 8:**
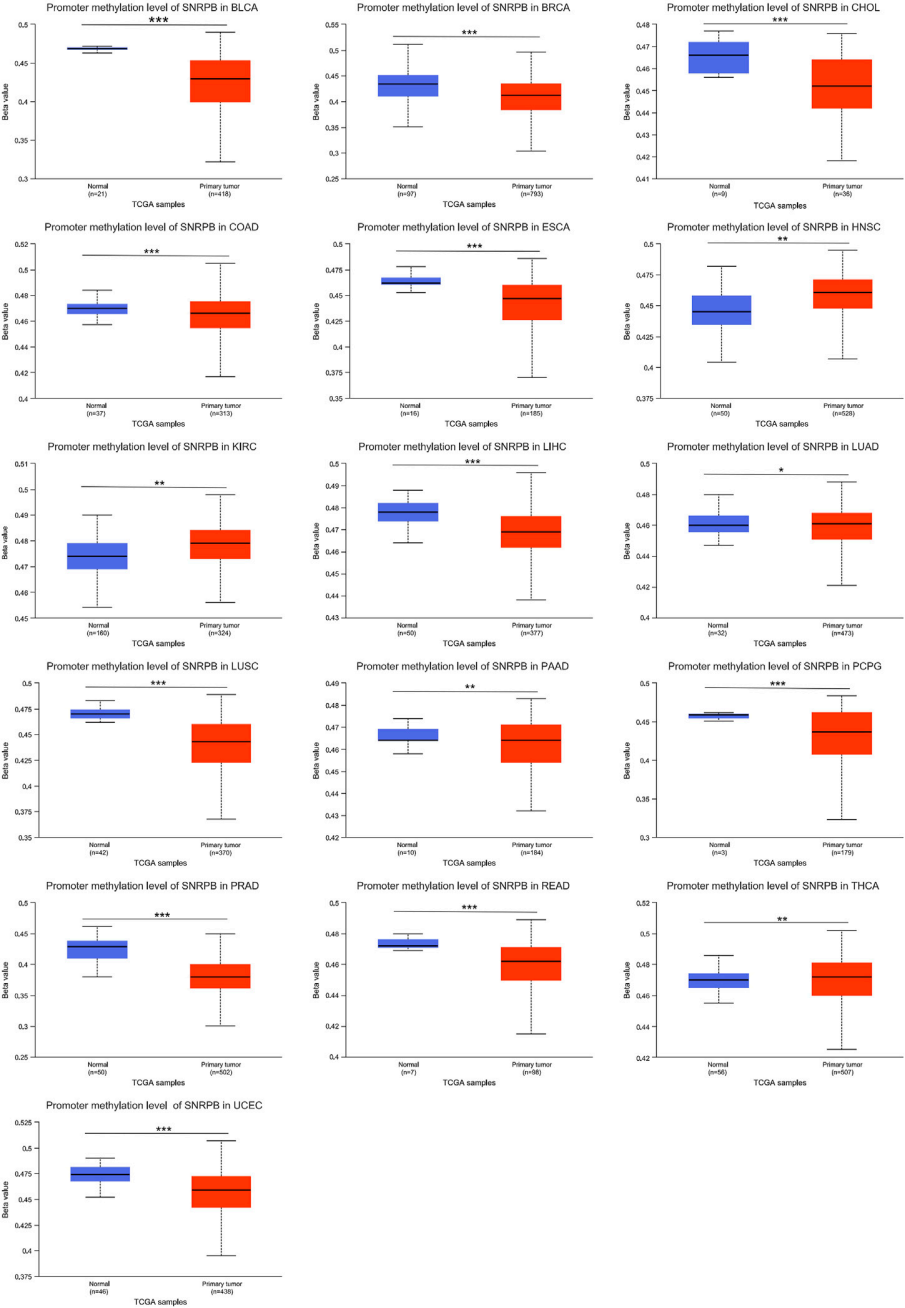
The promoter methylation level of SNRPB across tumors. *, *p* < 0.05; **, *p* < 0.01; ***, *p* < 0.001.

Methylation is one of the ways in which nucleobases are chemically modified, and genes can be silenced and reactivated by the methylation and demethylation of cytosines in the promoter region (Traube and Carell, 2017). To explore the mechanism of elevated SNRPB expression across tumors, we obtained data on SNRPB promoter methylation in the TCGA dataset from UALCAN. As shown in Figure 8, the SNRPB promoter methylation level was significantly altered in 16 tumors (*p* < 0.05), of which 12 tumors (including BLCA, BRCA, CHOL, COAD, ESCA, LIHC, LUSC, PAAD, PCPG, PRAD, READ, and UCEC) showed significantly decreased levels of SNRPB promoter methylation (*p* < 0.05). The above results suggest that the decreased methylation level of the SNRPB promoter may be the reason for its increased expression across tumors.

### Coexpressed genes and PPI network

**FIGURE 9:**
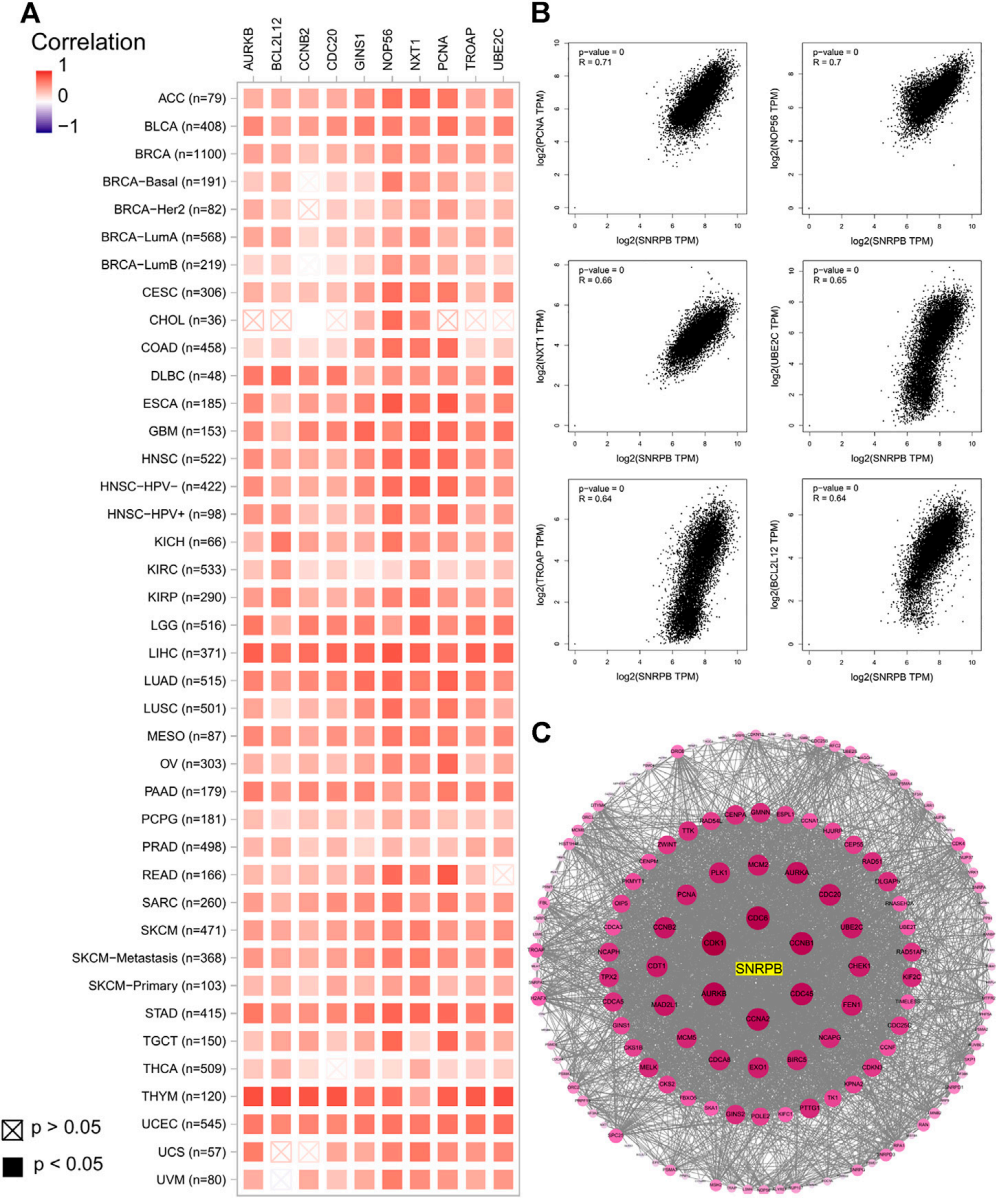
Coexpressed genes of SNRPB across tumors. **(A)**. Top 10 coexpressed genes of SNRPB across tumors. **(B)**. Representative figures of the relationship between SNRPB and the top 6 coexpressed genes across tumors. **(C)**. PPI network of SNRPB and the top 100 coexpressed genes across tumors.

We obtained the top 100 coexpressed genes from GEPIA2 to explore genes closely associated with SNRPB expression across tumors. The top 10 coexpressed genes were PCNA, NOP56, NXT1, UBE2C, TROAP, BCL2L12, GINS1, AURKB, CDC20, and CCNB2 (Figure 9A). We showed the relationship between SNRPB and the top 6 coexpressed genes across tumors in Figure 9B. Then, we used STRING and Cytoscape to plot the PPI network of SNRPB and the top 100 coexpressed genes. As shown in Figure 9C, CDK1, CDC6, AURKB, CCNB1, CCNA2, and CDC45 were the most closely interacting genes across tumors.

### Functional annotation

**FIGURE 10:**
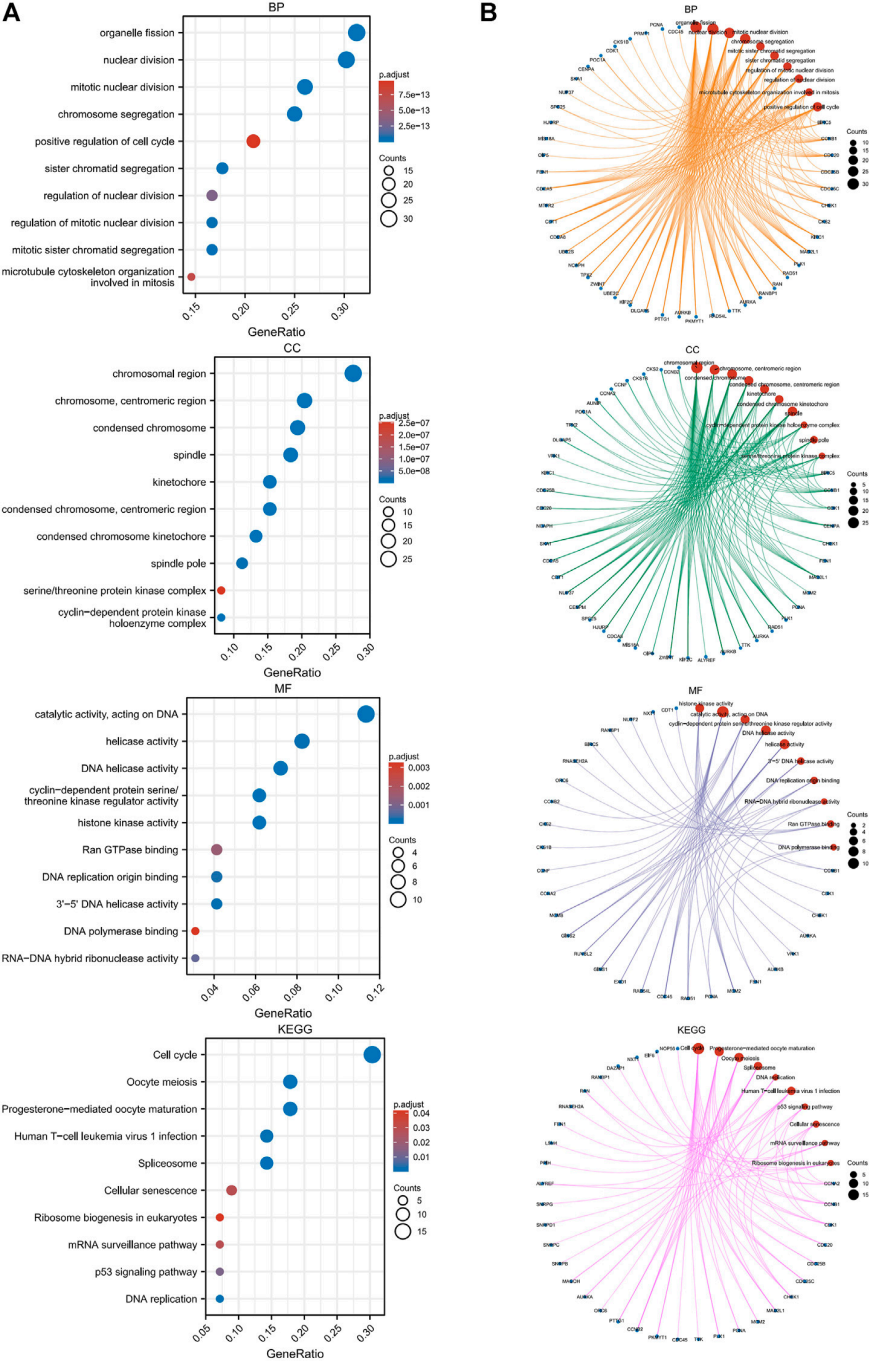
GO and KEGG enrichment analyses of SNRPB and the top 100 coexpressed genes across tumors. **(A)**. GO [in biological process (BP), cellular component (CC), molecular function (MF)] and KEGG enrichment analysis. **(B)**. Crosstalk between enriched BP, CC, MF, and KEGG functions and genes.

We performed GO and KEGG enrichment analyses of SNRPB and the top 100 coexpressed genes (101 genes in total) to explore the roles of these genes across tumors. As shown in Figure 10A, the most enriched biological processes (BP) were related to organelle fission, nuclear division, chromosome segregation, microtubule cytoskeleton organization involved in mitosis, and positive regulation of the cell cycle; the most enriched cellular components (CC) were related to the chromosomal region, centromeric region, kinetochore, spindle, cyclin-dependent protein kinase holoenzyme complex, and serine/threonine protein kinase complex; the most enriched molecular functions (MF) were related to catalytic activity, DNA replication origin binding, Ran GTPase binding, and DNA polymerase binding; and the most enriched KEGG pathways were related to cell cycle, oocyte maturation, spliceosome, DNA replication, human T-cell leukemia virus 1 infection, p53 signaling pathway, cellular senescence, mRNA surveillance pathway, and ribosome biogenesis in eukaryotes. The network plot showed the crosstalk between enriched BP, CC, MF, and KEGG functions and genes (Figure 10B). The above research results suggest that related genes are mainly enriched in the processes of cell cycle-related genetic material replication, assembly, and distribution, which indicates that the oncogenic role of SNRPB across tumors may be primarily achieved by affecting the cell cycle.

### Relationship between SNRPB and immune cell infiltration across tumors

**FIGURE 11:**
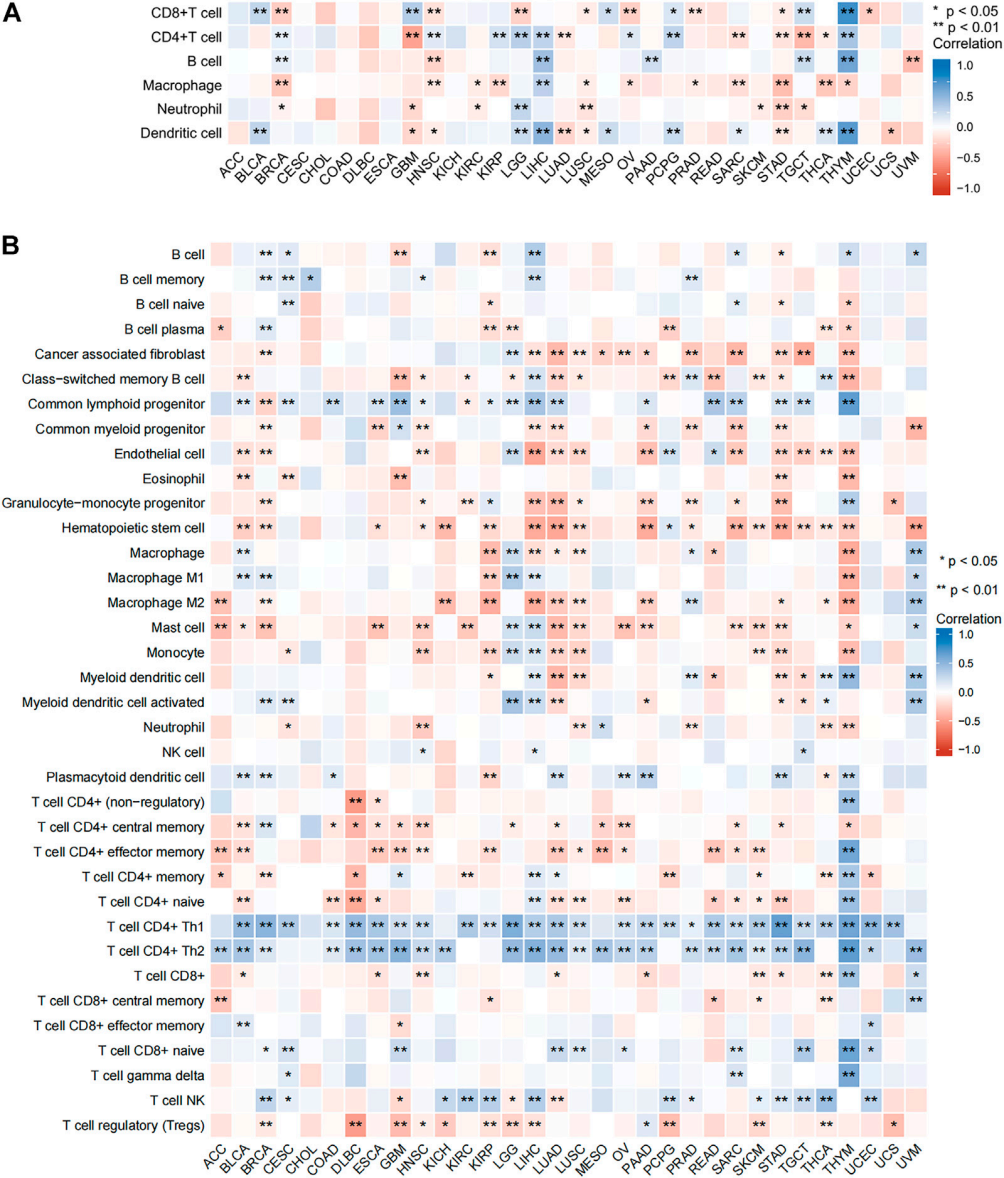
Relationship between SNRPB and immune cell infiltration. **(A)**. Relationship between SNRPB and immune cell infiltration based on the TIMER algorithm. **(B)**. Relationship between SNRPB and the infiltration of immune cell subtypes based on the xCell algorithm.

To further clarify the role of SNRPB across tumors, we assessed the relationship between SNRPB and tumor-infiltrating immune cells using TIMER2 (Figure 11). As shown in Figure 11A (TIMER algorithm, purity adjustment), SNRPB was significantly associated with immune infiltration across tumors, including CD8^+^ T cells, CD4^+^ T cells, and dendritic cells in 14 tumors, B cells in 7 tumors, macrophages in 12 tumors, and neutrophils in 8 tumors. We then used the xCell algorithm (purity adjustment) to evaluate the relationship between SNRPB and the infiltration of immune cell subtypes (Figure 11B). The results showed that SNRPB was significantly positively correlated with the infiltration of CD4+Th1 and CD4+Th2 cells across tumors.

### Relationship of SNRPB with the expression of immunomodulation-related genes across tumors

In order to further explore the oncogenic effects of SNRPB on tumor immune surveillance, immune escape, and immune infiltration, we downloaded data from TIMER2 pertaining to the relationship between SNRPB and the expression of immune activation-related, immunosuppression-related, chemokine, and chemokine receptor genes across tumors. As shown in Supplementary Figure S2, SNRPB was significantly positively correlated with the expression of almost all immune activation-related and immunosuppression-related genes in LGG, LIHC, and UVM and significantly negatively correlated in LUAD and LUSC. As shown in Supplementary Figure S3, SNRPB was significantly positively correlated with the expression of almost all chemokines and chemokine receptor genes in LGG, LIHC, and UVM and significantly negatively correlated in LUSC and thymoma (THYM). Moreover, SNRPB was also significantly negatively correlated with the expression of almost all chemokine receptor genes in LUAD, OV, PRAD, SKCM, STAD, UCEC, and uterine carcinosarcoma (UCS).

## Discussion

In the present study, we used multiple public database platforms to conduct an in-depth pan-cancer exploration of the role of SNRPB with respect to mRNA and protein expression, clinical outcome, genetic variation, promoter methylation, functional enrichment analysis, and tumor-infiltrating immune cells. Our results indicate that SNRPB expression was elevated in 28 tumors, correlated with late pathology stages and high TNM stages and was a risk factor for decreased OS, DSS, and PFI across tumors. Genetic variation in SNRPB may be associated with poor prognosis, and promoter methylation may be one of the mechanisms by which SNRPB was elevated across tumors. The PPI network revealed that CDK1, CDC6, AURKB, CCNB1, CCNA2, and CDC45 were the most closely interacting genes across tumors. Enrichment analysis of SNRPB and the most associated coexpressed genes were closely related to the cell cycle pathway. Moreover, SNRPB was also closely related to immune cell infiltration and the expression of immunomodulation-related genes in several tumors.

The pre-mRNA produced by DNA translation contains protein-coding exons and noncoding introns; introns are excised in the subsequent process, and exons are combined in different ways under the action of the spliceosome to produce various structurally and functionally distinct proteins (Sciarrillo et al., 2020). SNRPB is an important component of the spliceosome and thus is essential for the diversity of expressed proteins (Saltzman et al., 2008). New proteins produced by SNRPB dysregulation may be involved in tumorigenesis and progression (Correa et al., 2016; Liu et al., 2019; Peng et al., 2020; Zhan et al., 2020). Factors affecting gene expression levels include genetic alteration, epigenetic modifications, noncoding RNAs, mRNA transcriptional stability, and upstream transcription factors (Gu et al., 2017). Our results indicate that SNRPB expression was elevated in almost all tumors, and this change may be related to genetic alterations and decreased promoter methylation levels (epigenetic modifications). Posttranscriptional regulation is another important way to regulate gene expression, and microRNAs (miRNAs) are key posttranscriptional gene regulators (Cantini et al., 2019). Correa, B.R. et al. found that downregulation of tumor suppressor miRNAs could significantly trigger the overexpression of SNRPB in GBM (Correa et al., 2016). Another study in LIHC found that SNRPB is the downstream target of c-Myc, and the elevated expression of SNRPB was caused by overexpression of c-Myc (Peng et al., 2020). Therefore, the elevated expression of SNRPB in tumors may involve multiple mechanisms, including genetic alterations, epigenetic modifications, posttranscriptional modifications, and changes in the expression levels of upstream molecules.

Proliferation and migration are the basis of the progression and metastasis of tumors (Bucay et al., 2017). Both *in vitro* and *in vivo* experiments show that SNRPB can significantly promote the proliferation and migration of various tumor cells. *In vitro* experiments showed that overexpression of SNRPB could significantly promote cell growth, the formation of microspheres, and the migration of cells, while silencing SNRPB reduced proliferation, colony formation, and the number of migrated cells (Peng et al., 2020; Zhan et al., 2020). A similar phenomenon has been observed in NSCLC cells (Liu et al., 2019), CESC cells (Zhu et al., 2020), GBM cells (Correa et al., 2016), and THCA cells (Deng et al., 2022). A xenograft tumor assay indicated that transplanted tumors derived from SNRPB-transfected cells exhibit larger volumes and higher weights, and knockdown of SNRPB inhibits tumor growth (Zhan et al., 2020). The effect of SNRPB on the invasion, metastasis, and progression of tumors has also been confirmed (Liu et al., 2019; Zhu et al., 2020). The results of our study indicate that SNRPB can promote the progression of pathological and TNM stages, which is supported by the above studies.

The ultimate goal of oncology research is to cure the disease, improve the quality of life and prolong survival (Ogino et al., 2011). Therefore, the survival of patients is an important prognostic indicator. Our study found that SNRPB was a risk factor for decreased OS in 10 tumors, decreased DSS in 8 tumors, and decreased PFI in 7 tumors, suggesting that SNRPB could be a prognostic factor across tumors. At present, almost all studies on the effect of SNRPB on tumor prognosis are conducted based on public databases (Liu et al., 2019; Peng et al., 2020; Zhan et al., 2020). The basic findings provide clues regarding the mechanisms of the adverse effects of SNRPB on tumor prognosis. SNRPB not only promotes tumor proliferation, migration, and metastasis but also maintains the stemness of tumor cells (Zhan et al., 2020) and affects tumor responsiveness to therapy (Liu et al., 2021). Therefore, SNRPB may be involved in many aspects, such as tumorigenesis, progression, metastasis, maintenance of stemness, and treatment responsiveness, acting with adverse effects on the prognosis of tumors.

Cancer is a disease with extremely complex mechanisms and phenotypes. During the process of transforming normal cells into cancer cells, cells acquire shared functions necessary for their malignancy, such as sustained proliferation (Hanahan, 2022). Sustained proliferation represents the continuous entry of cells into the cell cycle, which means sustained activation of the cell cycle pathway. The cell cycle is a collection of events involving the replication of genetic material and distribution of replicated genetic material and cytoplasmic components to daughter cells (Zou and Lin, 2021). Through the PPI network, we identified the most closely interacting genes across tumors, including CDK1, CDC6, AURKB, CCNB1, CCNA2, and CDC45. All of these identified genes are associated with cell cycle processes such as cell cycle transitions, DNA replication, DNA damage, and mitosis. KEGG enrichment analysis revealed that the cell cycle was the most enriched pathway, while GO enrichment analysis revealed that almost all enriched BP, CC, and MF functions were related to the cell cycle. These results indicate that the oncogenic role of SNRPB across tumors may be mainly achieved by affecting the cell cycle, and it would be very interesting to investigate the effect of SNRPB on cell cycle-targeted therapies across tumors in the future. AS is an important way to regulate gene expression and protein diversity, and 95% of multiexon transcripts undergo AS (Pan et al., 2008). The spliceosome pathway, which was significantly enriched in KEGG enrichment analysis, is required for AS. p53 is a well-established tumor suppressor gene, and the loss of p53 function is associated with tumor progression (Hanahan, 2022). Cellular senescence has long been recognized as a tumor suppression mechanism (Lee and Schmitt, 2019); however, recent studies have found that senescent cancer cells can promote proliferative signals, avoid apoptosis, induce angiogenesis, stimulate invasion and metastasis, and suppress tumor immunity in different ways (He and Sharpless, 2017; Faget et al., 2019; Lee and Schmitt, 2019; Wang et al., 2020). The p53 signaling pathway and cellular senescence were also significantly enriched in KEGG enrichment analysis, and the inhibitory effect of SNRPB on the p53 signaling pathway has been confirmed in CESC (Zhu et al., 2020). Therefore, we speculate that the oncogenic role of SNRPB across tumors may be mainly achieved by affecting cell cycle-related processes. In addition, the spliceosome, p53 signaling pathway, and cell senescence may also contribute to its oncogenic effect.

The tumor microenvironment (TME) is an integral part of cancer that significantly affects treatment response and clinical outcomes. As part of the TME, immune cells exert important impacts on tumor progression and prognosis (Pitt et al., 2016). Our study found that SNRPB was strongly associated with immune cell infiltration in some tumors and to a lesser extent or not at all in others. Immune cell subtype analysis revealed that SNRPB was significantly positively correlated with the infiltration of CD4+Th1 and CD4+Th2 in almost all tumors. These results suggest that the relationship between SNRPB and the infiltration of immune cells other than CD4+Th1 and CD4+Th2 cells is tumor specific, and this specificity may be related to the effect of SNRPB on the expression of immunomodulation-related genes. At present, there are few studies on the relationship between SNRPB and tumor immune cell infiltration, and more research is needed to confirm the relationship and mechanism in the future.

Here, for the first time, we systematically analyzed SNRPB expression, genetic variation, promoter methylation, and the relationship with prognosis, immune cell infiltration, and immunomodulation-related genes across tumors and preliminarily explored the oncogenic effect and mechanism of SNRPB. However, our study also has some limitations. First, our research was based on data obtained from public databases, without further validation of our findings at the cellular and animal levels. Furthermore, we failed to systematically and deeply explore the pathophysiological mechanisms underlying the results. Therefore, further studies are needed to clarify the oncogenic mechanism of SNRPB and its potential as a therapeutic target.

## Conclusion

The expression of SNRPB was significantly elevated in almost all tumors, and the decreased promoter methylation level may contribute to the elevated expression of SNRPB. SNRPB may facilitate the progression of pathological and TNM stages and is a risk factor for unfavorable prognosis across tumors.

## Data Availability

The datasets presented in this study can be found in online repositories. The names of the repository/repositories can be found in the article/Supplementary Material.

## Supplementary material

Supplementary Figure 1 validation of SNRPB expression in tumors and the corresponding normal tissues using data from the GEO database. ns, no significant difference; ^*^ *p* < 0.05; ^**^ *p* < 0.01; ^***^ *p* < 0.001.

Supplementary Figure 2 Relationship of SNRPB with the expression of immunomodulation-related genes across tumors. **(A)** The relationship of SNRPB and the expression of immune activation-related genes across tumors. **(B)** The relationship of SNRPB and the expression of immunosuppression-related genes across tumors.

Supplementary Figure 3 Relationship of SNRPB with the expression of immunomodulation-related genes across tumors. **(A)** The relationship of SNRPB and the expression of chemokine genes across tumors. **(B)** The relationship of SNRPB and the expression of chemokine receptor genes across tumors.
