# Supplementary figures and images for: The oncogenic role of SNRPB in human tumors: A pan-cancer analysis
Source: Front Mol Biosci. 2022 Oct 6;9:994440. doi: 10.3389/fmolb.2022.994440 (PMC9582665; doi:10.3389/fmolb.2022.994440)

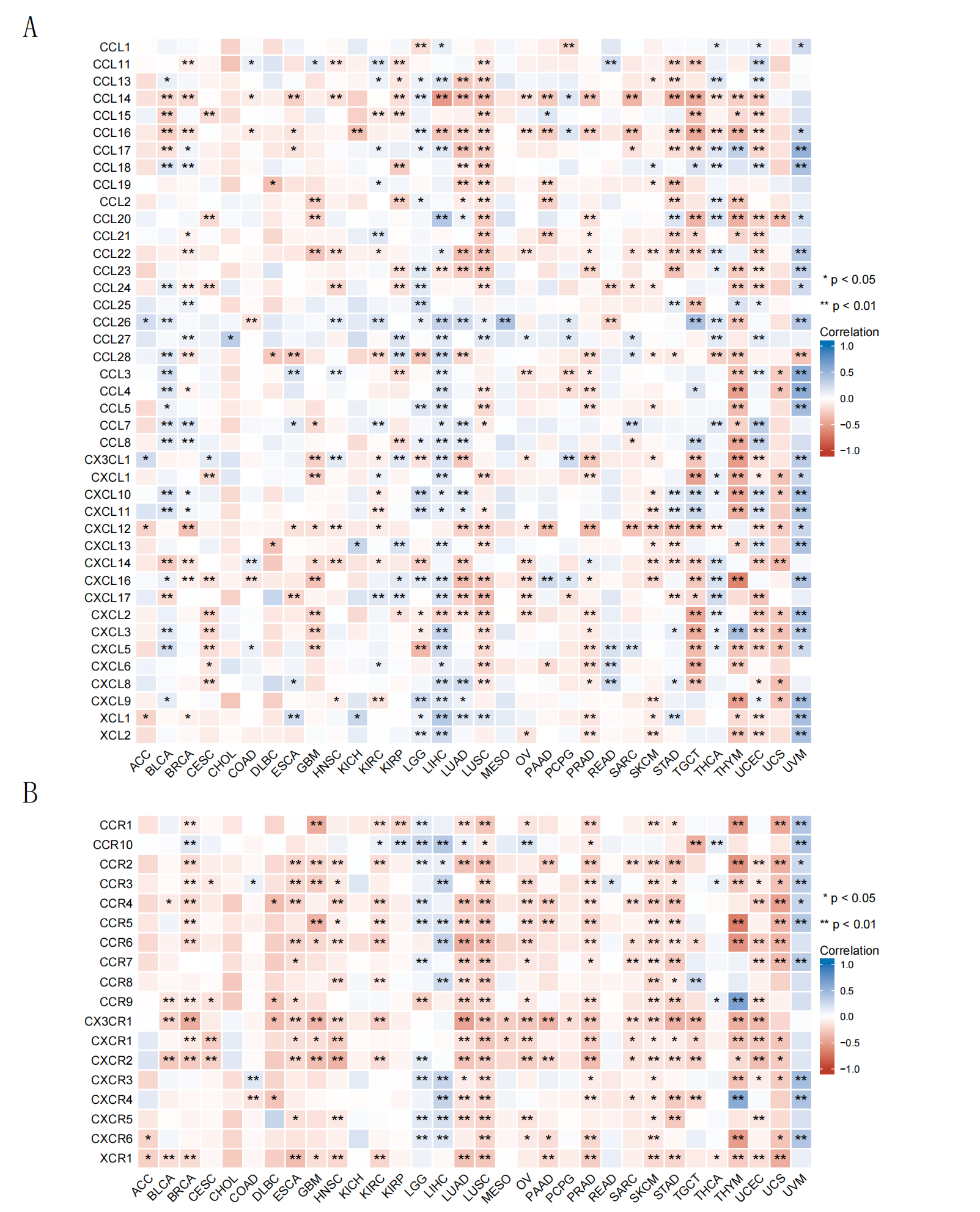

Supplement: Supplementary file 1 [file Image3.TIF]

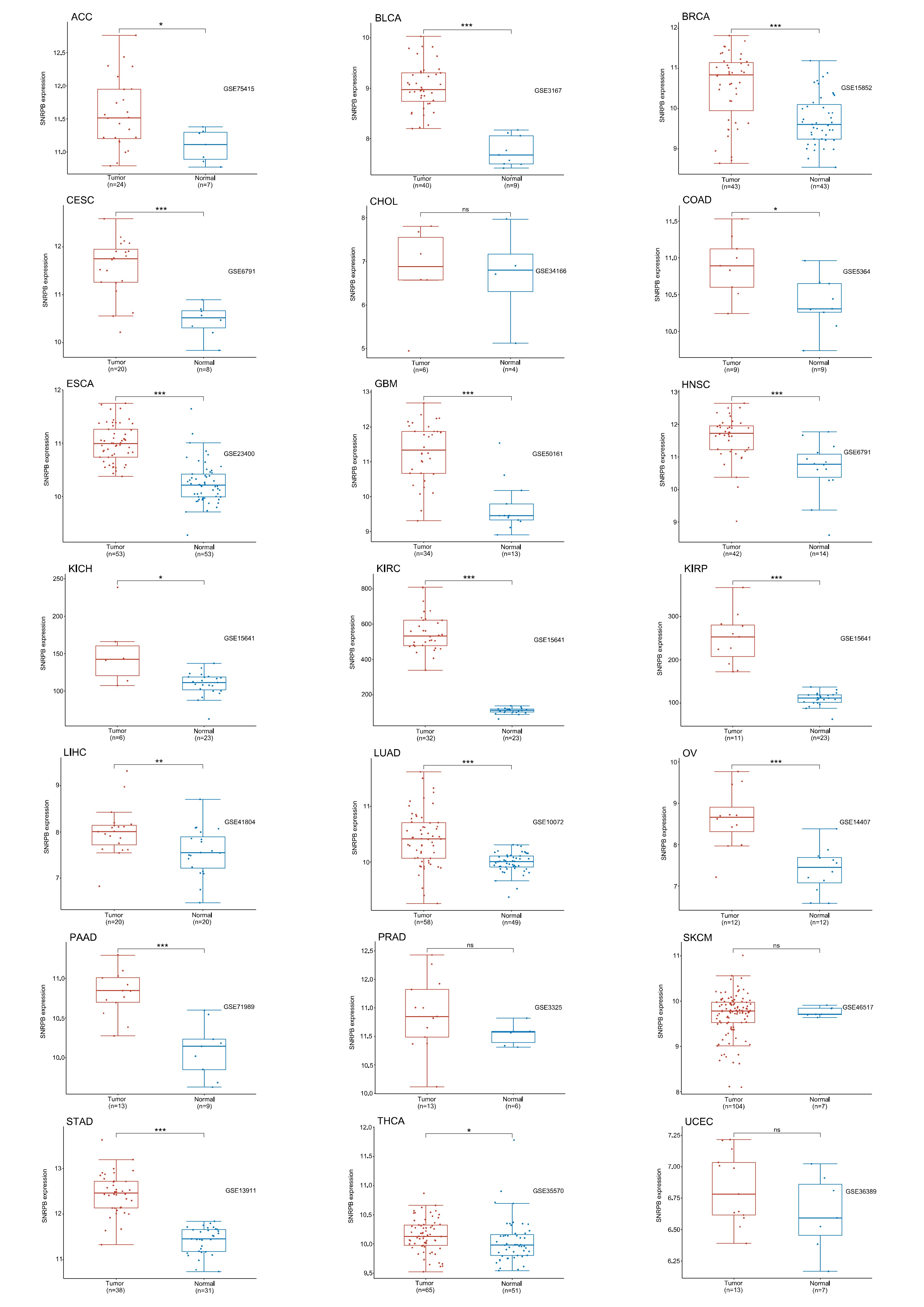

Supplement: Supplementary file 2 [file Image1.JPEG]

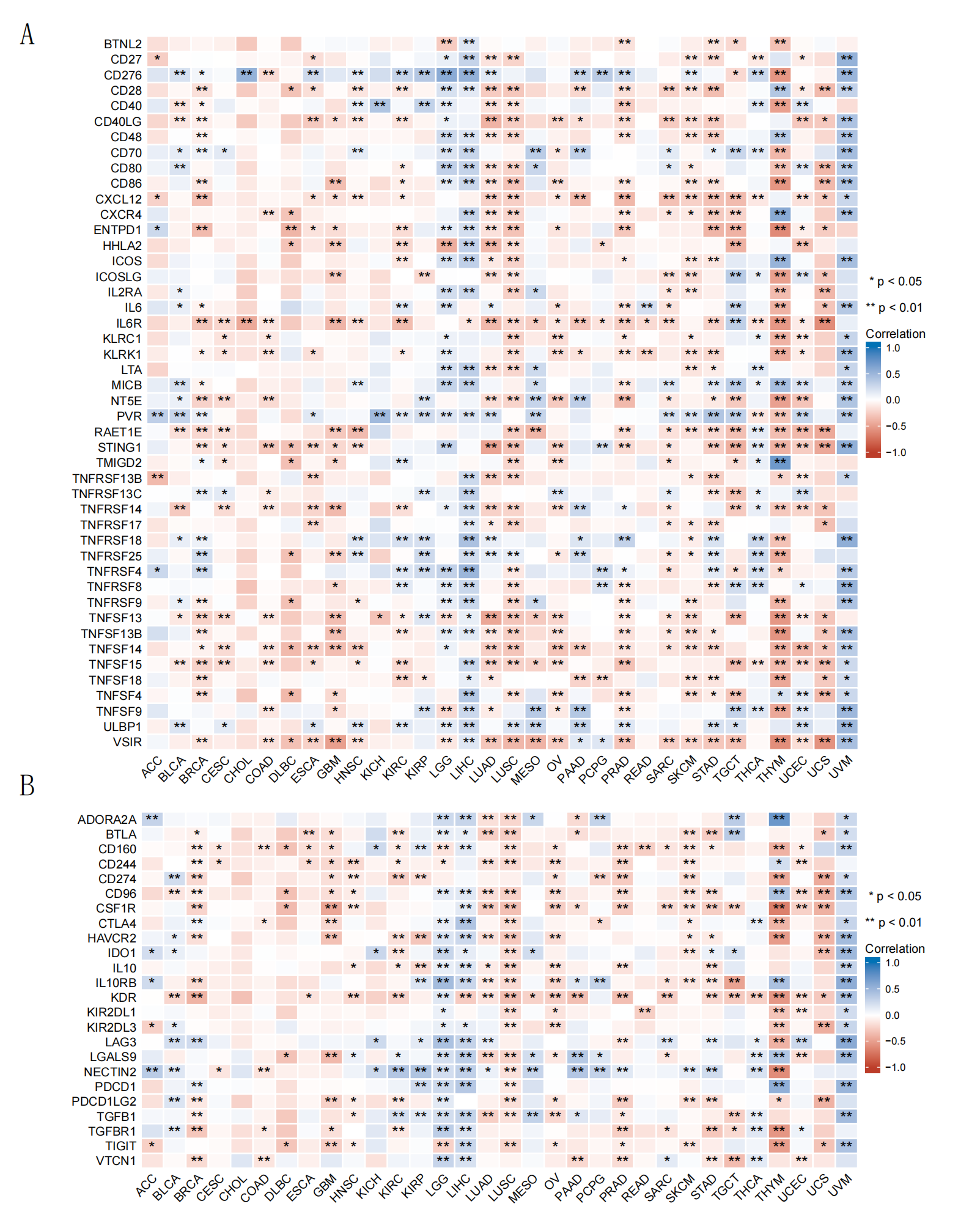

Supplement: Supplementary file 3 [file Image2.TIF]
